# Sitagliptin use and thyroid cancer risk in patients with type 2 diabetes

**DOI:** 10.18632/oncotarget.8399

**Published:** 2016-03-28

**Authors:** Chin-Hsiao Tseng

**Affiliations:** ^1^ Department of Internal Medicine, National Taiwan University College of Medicine, Taipei, Taiwan; ^2^ Division of Endocrinology and Metabolism, Department of Internal Medicine, National Taiwan University Hospital, Taipei, Taiwan; ^3^ Division of Environmental Health and Occupational Medicine of the National Health Research Institutes, Zhunan, Taiwan

**Keywords:** thyroid cancer, diabetes mellitus, sitagliptin, Taiwan

## Abstract

Whether sitagliptin may increase thyroid cancer risk has not been investigated in the Asian populations. This study evaluated the association in Taiwanese patients with newly diagnosed type 2 diabetes from 1999 to 2008 by using the reimbursement database of the National Health Insurance. They should have been followed for at least 6 months after March 1, 2009, the date when sitagliptin was approved for reimbursement. Patients newly treated with sitagliptin (*n*=58238, “ever users of sitagliptin”) or other antidiabetic drugs (*n* =312853, “never users of sitagliptin”) were followed until December 31, 2011. The treatment effect (for ever versus never users, and for tertiles of cumulative duration of therapy) was estimated by Cox regression incorporated with the inverse probability of treatment weighting using propensity score. Results showed that the respective number of incident thyroid cancer in ever users and never users was 28 and 172, with respective incidence of 29.34 and 22.13 per 100,000 person-years. The overall hazard ratio (95% confidence interval) of 1.516 (1.011-2.271) suggested a significantly higher risk associated with sitagliptin use. In tertile analyses, the hazard ratio for the first (< 6.53 months), second (6.53-14.00 months) and third (> 14 months) tertile of cumulative duration was 1.995 (1.015-3.919), 2.516 (1.451-4.364) and 0.595 (0.244-1.449), respectively. Analyses after excluding patients with benign thyroid disease and in a subsample matched on baseline characteristics supported the findings in the original sample. In conclusion, sitagliptin use is associated with an increased risk of thyroid cancer, especially during the first year of its treatment.

## INTRODUCTION

Incretin-based therapies in patients with type 2 diabetes mellitus by using either the injection form of glucagon-like peptide-1 receptor (GLP-1R) agonists or the oral form of dipeptidyl peptidase-4 (DPP-4) inhibitors have been shown to increase the risk of thyroid cancer [1, 2]. Elashoff et al. [3] analyzed the database of the US Food and Drug Administration (FDA) adverse event reporting system. They showed that the odds ratio (OR) for exenatide (a GLP-1R agonist) was 4.73 (*P* = 4 × 10^−3^); and for sitagliptin (a DPP-4 inhibitor) 1.48 (*P* = 0.65). An updated analysis by the same group concluded that GLP-1R agonists of exenatide and liraglutide were associated with a significantly higher risk, with respective OR (95% confidence interval, CI) of 3.94 (2.56-6.20) and 17.99 (10.12-33.56). However the estimated OR (95% CI) for sitagliptin [1.08 (0.33-2.81)] attenuated and was not significant. No case of thyroid cancer was observed for other DPP-4 inhibitors such as saxagliptin and linagliptin [4]. Therefore, the risk of thyroid cancer associated with incretin-based therapies is controversial and may differ between GLP-1R agonists and DPP-4 inhibitors or among different DPP-4 inhibitors.

Whether sitagliptin may increase the risk of thyroid cancer has not been studied in the Asian populations. The present study evaluated such a risk association in Taiwanese patients by using the reimbursement records of the National Health Insurance (NHI) databases.

## RESULTS

**Figure 1:**
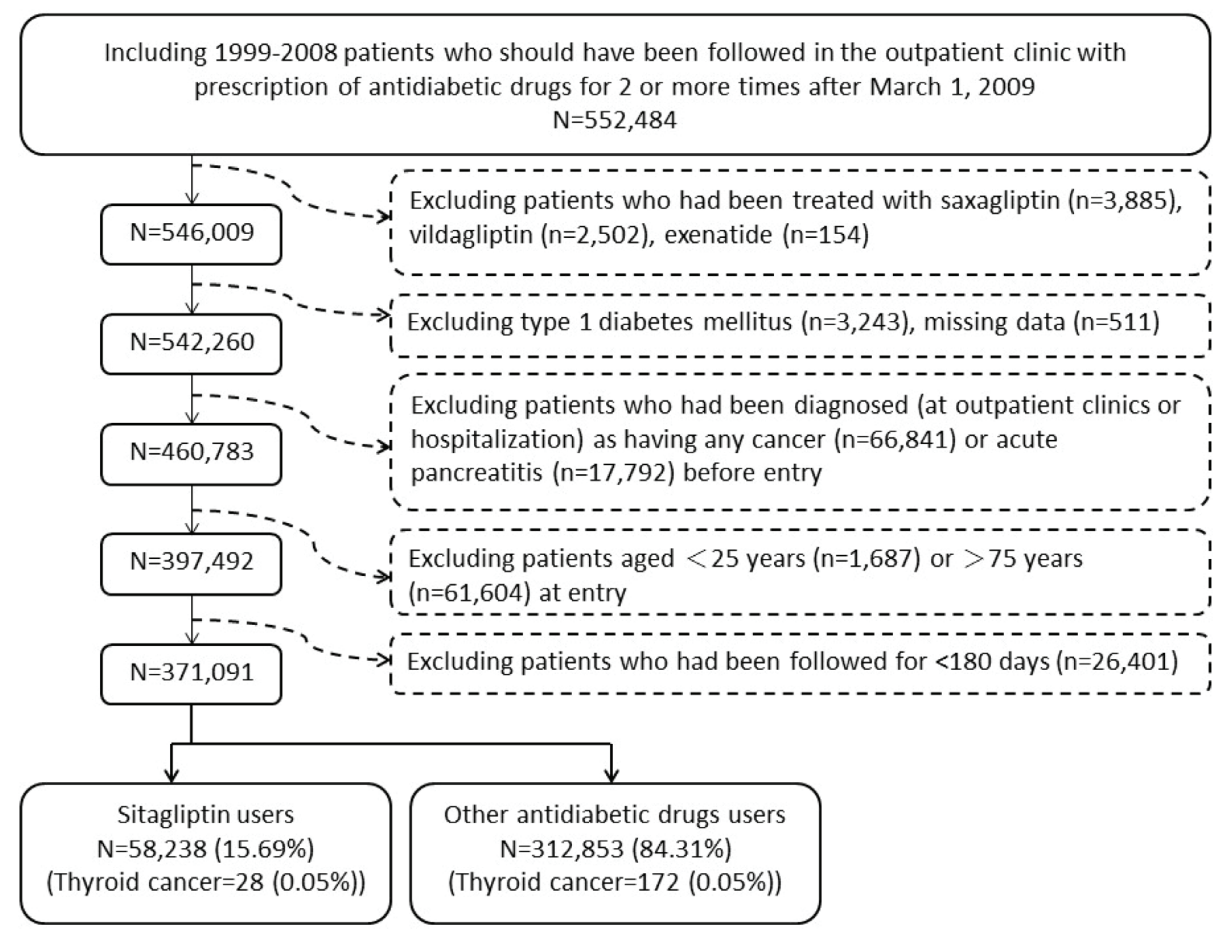
Flowchart showing the procedures in selecting the original sample into the study

There were 312853 never users and 58238 ever users in the original sample (Figure 1). All characteristics differed significantly, except for chronic obstructive pulmonary disease (Table 1). Ever users were characterized by younger age, longer diabetes duration, less males, higher proportions of all comorbidities, less proportion of sulfonylurea but higher proportions of other antidiabetic medications (Table 1). In the matched sample, baseline characteristics were more comparable and only 4 variables (age, diabetes duration, sulfonylurea, and acarbose) remained significantly different. Eight out of the 21 variables had standardized differences > 10% in the original sample, but none had a value > 10% in the matched sample (Table 1).

**Table 1:** Comparison of baseline characteristics between sitagliptin never users and ever users in the original sampleand in the propensity score matched sample

Variable	Original sample						Matched sample					
	Never users		Ever users				Never users		Ever users			
	(*n* = 312853)		(*n* = 58238)		P*	SD	(*n* = 57659)		(*n* = 57659)		P*	SD
	Mean ± standard deviation		Mean ± standard deviation				Mean ± standard deviation		Mean ± standard deviation			
Age (years)	57.7±10.0		57.0±10.0		<0.0001	−10.57	56.9±10.1		57.0±9.9		0.0083	1.53
Diabetes duration (years)	5.4±2.9		7.0±2.8		<0.0001	61.10	7.0±2.7		7.0±2.8		0.0164	1.03
	*n*	%	*n*	%			*n*	%	*n*	%		
Sex												
Women	142985	45.70	27227	46.72	<0.0001	−1.97	27094	46.99	26943	46.73	0.3729	0.52
Men	169868	54.30	31011	53.21			30565	53.01	30716	53.27		
Hypertension												
No	84776	27.10	13365	22.93	<0.0001	9.31	13392	23.23	13301	23.07	0.5252	0.31
Yes	228077	72.90	44873	76.99			44267	76.77	44358	76.93		
Chronic obstructive pulmonary disease												
No	183696	58.72	33955	58.26	0.0634	0.21	33626	58.32	33607	58.29	0.9097	0.05
Yes	129157	41.28	24283	41.66			24033	41.68	24052	41.71		
Heart failure												
No	277100	88.57	50045	85.87	<0.0001	7.65	49627	86.07	49625	86.07	0.9864	−0.03
Yes	35753	11.43	8193	14.06			8032	13.93	8034	13.93		
Nephropathy												
No	257388	82.27	45877	78.71	<0.0001	9.06	45705	79.27	45481	78.88	0.1049	0.92
Yes	55465	17.73	12361	21.21			11954	20.73	12178	21.12		
Eye disease												
No	262014	83.75	41320	70.90	<0.0001	27.97	41358	71.73	41257	71.55	0.5093	0.32
Yes	50839	16.25	16918	29.03			16301	28.27	16402	28.45		
Dyslipidemia												
No	80344	25.68	10566	18.13	<0.0001	20.60	10417	18.07	10554	18.30	0.2956	−0.65
Yes	232509	74.32	47672	81.79			47242	81.93	47105	81.70		
Stroke												
No	248921	79.56	44946	77.12	<0.0001	5.78	44495	77.17	44535	77.24	0.7789	−0.21
Yes	63932	20.44	13292	22.81			13164	22.83	13124	22.76		
Ischemic heart disease												
No	200694	64.15	34614	59.39	<0.0001	9.63	34411	59.68	34372	59.61	0.8149	0.11
Yes	112159	35.85	23624	40.53			23248	40.32	23287	40.39		
Peripheral arterial disease												
No	258381	82.59	46610	79.97	<0.0001	6.14	46096	79.95	46179	80.09	0.5410	−0.38
Yes	54472	17.41	11628	19.95			11563	20.05	11480	19.91		
Obesity												
No	297731	95.17	54005	92.66	<0.0001	10.38	53402	92.62	53563	92.90	0.0674	−1.12
Yes	15122	4.83	4233	7.26			4257	7.38	4096	7.10		
Benign thyroid disease												
No	280136	89.54	50903	87.34	<0.0001	8.32	50381	87.38	50453	87.50	0.5223	−0.43
Yes	32717	10.46	7335	12.59			7278	12.62	7206	12.50		
Sulfonylurea												
No	111828	35.74	23575	40.45	<0.0001	−13.42	23630	40.98	23193	40.22	0.0088	1.69
Yes	201025	64.26	34663	59.47			34029	59.02	34466	59.78		
Metformin												
No	85925	27.46	14148	24.27	<0.0001	11.96	13834	23.99	14001	24.28	0.2505	−0.74
Yes	226928	72.54	44090	75.65			43825	76.01	43658	75.72		
Meglitinide												
No	298548	95.43	54885	94.17	<0.0001	5.51	54328	94.22	54372	94.30	0.5775	−0.31
Yes	14305	4.57	3353	5.75			3331	5.78	3287	5.70		
Acarbose												
No	288189	92.12	51120	87.71	<0.0001	14.38	50537	87.65	50800	88.10	0.0177	−1.44
Yes	24664	7.88	7118	12.21			7122	12.35	6859	11.90		
Insulin												
No	297534	95.10	54769	93.97	<0.0001	7.79	54179	93.96	54218	94.03	0.6287	−0.31
Yes	15319	4.90	3469	5.95			3480	6.04	3441	5.97		
Pioglitazone												
No	295794	94.55	54174	92.95	<0.0001	8.52	53704	93.14	53658	93.06	0.5930	0.29
Yes	17059	5.45	4064	6.97			3955	6.86	4001	6.94		
Rosiglitazone												
No	304161	97.22	56349	96.68	<0.0001	5.94	55762	96.71	55782	96.74	0.7406	−0.18
Yes	8692	2.78	1889	3.24			1897	3.29	1877	3.26		

* Chi square test for age and diabetes duration, and Student's t test for other variables SD: standardized difference

Table 2 shows the incidence of thyroid cancer with regards to sitagliptin. The respective number of incident thyroid cancer for never users and ever users in the original sample was 172 and 28, with respective incidence of 22.13 and 29.34 per 100,000 person-years. The incidences for users within the first year (in the first and second tertiles) were higher than never users but the incidence was lower for users of more than one year (third tertile) when compared to never users. Findings in the matched sample were similar to those observed in the original sample.

**Table 2:** Incidence of thyroid cancer by sitagliptin exposure

Sitagliptin use	Case number	Incident thyroid cancer	%	Person-years	Incidence rate
					(per 100,000 person-years)
I. Original sample					
Never users	312853	172	0.05	777260.24	22.13
Ever users	58238	28	0.05	95424.71	29.34
**Tertiles of cumulative duration of sitagliptin therapy (months)**					
Never users	312853	172	0.05	777260.24	22.13
<6.53	18287	9	0.05	25343.96	35.51
6.53-14.00	20161	14	0.07	29760.78	47.04
>14	19790	5	0.03	40319.97	12.40
II. Matched sample					
Never users	57659	31	0.05	141851.35	21.85
Ever users	57659	28	0.05	94698.12	29.57
**Tertiles of cumulative duration of sitagliptin therapy (months)**					
Never users	57659	31	0.05	141851.35	21.85
<6.53	18066	9	0.05	25104.96	35.85
6.53-14.00	19978	14	0.07	29578.69	47.33
>14	19615	5	0.03	40014.47	12.50

Table 3 shows the hazard ratios in different groups of sitagliptin use. Although not all analyses were significant, the overall hazard ratios suggested a higher risk associated with sitagliptin use. When analyzed by the tertiles of cumulative duration, a significantly increased risk could be observed for the first and second tertiles, and the risk became neutral when sitagliptin use was > 14 months in the third tertile. When the hazard ratios were estimated in subgroups of metformin use, a significantly higher risk for sitagliptin ever users *versus* never users could only be observed among metformin users but not in non-users.

**Table 3:** Sitagliptin exposure and hazard ratios for thyroid cancer

Sitagliptin use/subgroups of metformin use	All patients			Excluding patients with benign thyroid disease		
	HR	95% CI	*P*	HR	95% CI	*P*
I. Original sample						
Ever users	1.516	(1.011-2.271)	0.0439	1.623	(0.978-2.694)	0.0612
**Tertiles of cumulative duration of sitagliptin therapy (months)**						
<6.53	1.995	(1.015-3.919)	0.0451	2.582	(1.193-5.591)	0.0161
6.53-14.00	2.516	(1.451-4.364)	0.0010	2.358	(1.141-4.872)	0.0205
>14	0.595	(0.244-1.449)	0.2530	0.590	(0.187-1.862)	0.3681
**Subgroups of metformin use**						
Metformin non-users	1.058	(0.418-2.680)	0.9045	1.105	(0.333-3.663)	0.8709
Metformin users	1.675	(1.067-2.630)	0.0251	1.792	(1.021-3.145)	0.0420
II. Matched sample						
Ever users	1.462	(0.866-2.466)	0.1551	1.597	(0.829-3.077)	0.1617
**Tertiles of cumulative duration of sitagliptin therapy (months)**						
<6.53	1.936	(0.906-4.137)	0.0881	2.725	(1.126-6.595)	0.0262
6.53-14.00	2.301	(1.208-4.386)	0.0113	2.261	(0.978-5.225)	0.0563
>14	0.574	(0.222-1.483)	0.2516	0.557	(0.164-1.886)	0.3469
**Subgroups of metformin use**						
Metformin non-users	0.912	(0.288-2.882)	0.8747	1.005	(0.224-4.512)	0.9949
Metformin users	1.662	(0.923-2.993)	0.0907	1.789	(0.863-3.710)	0.1178

Cox regression models were created by incorporation with the inverse probability of treatment weighting using propensity score created from variables in Table 1.

Referent group: never users of sitagliptin

HR: hazard ratio, CI: confidence intervals

Table 4 shows the prevalence of performance of thyroid sonography/aspiration examinations between sitagliptin ever users and never users. Never users of sitagliptin had a significantly higher rate of receiving these examinations.

**Table 4:** Performance of thyroid sonography/aspiration with regards to sitagliptin use

Sitagliptin	Cases receiving thyroid sonography/aspiration				*P*
	No		Yes		
	*n*	%	*n*	%	
I. Original sample					
Never users	307975	98.44	4878	1.56	<0.0001
Ever users	57529	98.78	709	1.22	
II. Matched sample					
Never users	56715	98.36	944	1.64	<0.0001
Ever users	56960	98.79	699	1.21	

## DISCUSSION

The findings suggested a significantly higher risk of thyroid cancer associated with sitagliptin use in the overall analyses comparing ever to never users in the original sample. However, the increased risk could only be observed in the first year of its use (Table 3). Because the baseline characteristics between sitagliptin ever and never users differed significantly in the original sample (Table 1), analyses were also conducted in a well-matched sample. Results derived from this well-matched sample were supportive for the findings in the original sample (Tables 2 and 3).

The studies analyzing the US FDA adverse event reporting system did not consistently find a significantly higher risk of thyroid cancer associated with sitagliptin [3, 4]. In the first analysis a higher risk was observed (though not statistically significant, OR = 1.48, *P* = 0.65) [3], but the estimated OR (1.08, 95% CI: 0.33-2.81) in the updated analysis suggested a null association [4]. The inconsistent findings during two different periods may be due to inherent limitations related to the use of the adverse event reporting system. These may include a lack of appropriate adjustment for confounders, channelling bias, disproportionate reporting, Weber effect and detection bias [2]. However, it is worthy to note that the 50% higher risk in the first analysis of the FDA database [3] was very close to the 50-60% increased risk in the present study (Table 3). If the increased risk could only be seen during the first year of sitagliptin use as shown in the present study (Table 3), it would not be surprising to see an increased risk in the first analysis of the FDA database and the null association in the second analysis due to the following reasons. During the period of the first analysis, many cases of sitagliptin-related thyroid cancer could have occured when sitagliptin have just been introduced into the market. In the second analysis, the reported cases of thyroid cancer would reduce because many patients have used sitagliptin for more than one year.

A treatment duration of one year might be too short to drive normal follicular cells to malignant change. Therefore, it would be interesting to explain why the risk of thyroid cancer increased only in the first year of sitagliptin use but reduced thereafter (Table 3). GLP-1R is expressed in all types of thyroid cells including normal, premalignant or malignant tissues [5-7]. Although Pyke et al. could not similarly demonstrate GLP-1R in normal thyroid tissues in either monkeys or humans by using a new monoclonal antibody [8], this could not exclude the expression of GLP-1R in pre-malignant and malignant thyroid cells. Stimulation of preexisting premalignant lesions or occult cancer to more rapidly progressive malignancy could be a possible explanation. Although the findings after excluding patients with benign thyroid disease might still indicate a risk association (Table 3), it should be noticed that the study could not exclude patients with undiagnosed subclinical thyroid nodular disease, which is very common in the general population.

On the other hand, with a longer duration of use, sitaglipitn might have exerted other beneficial effects that lead to a reduced risk of thyroid cancer. Recent clinical studies suggested that long-term use of sitagliptin for one year [9] or two years [10] may significantly reduce insulin resistance, an important risk factor for thyroid cancer [11]. The duration of at least one-year use of sitagliptin for reducing insulin resistance [9, 10] corresponded to the time frame for risk attenuation of thyroid cancer after one year of sitagliptin administration in the present study (Table 3). In addition, sitagliptin shows anti-inflammatory effects [12], which may also counteract partly its procancer effect. Therefore, the mechanisms leading to an increased risk of thyroid cancer during the early phase of sitagliptin use might have been counteracted by its long-term effect on the improvement of insulin resistance and inflammation. Whether an even longer duration of sitagliptin use may reduce the risk of thyroid cancer awaits further investigation.

The increased risk associated with sitagliptin use was mainly observed in metformin users but not in non-users (Table 3). The real reasons for such differential effects require further in-depth investigation. However, there are some possible explanations. First, metformin is usually used as a first-line therapy, therefore most of the patients were being treated with metformin when sitagliptin was added. Second, metformin is contraindicated in patients with abnormal renal function or those who were at risk of lactic acidosis, and gastrointestinal side effects can refrain some patients from being treated with metformin. Therefore, indication bias might exist between patients using and not using metformin. Third, the cumulative duration and dose of metformin treatment were not considered and metformin was not treated as a time-varying variable.

If prevalent users had been used in the study, it is possible that sitagliptin users represented those who survived with a less severe clinical disease and the propensity to develop cancer in these patients might not be similar to non-users of sitagliptin. Because the present study included only incident cases of diabetes and new-users of sitagliptin, such a potential “prevalent user bias” [13] should have been minimized. Additionally, the PS-weighted models were created to minimize the confounding of baseline subject characteristics associated with treatment allocation commonly seen in observational studies [14]. The exclusion of patients followed for < 6 months reduced the possibility of “immortal time bias” [15]. The consistency in the original sample and the well-matched sample (Tables 2 and 3) also suggested the reproducibility of the findings. The higher rates of receiving thyroid sonography/aspiration in never users of sitagliptin (Table 4) indicated that if detection bias did exist, this could only overestimate the incidence among never users leading to an underestimation of the hazard ratio comparing ever to never users.

This study has several strengths. First, the NHI databases included all longitudinal claims records, and we caught the diagnoses from all sources. Second, patients with a certified diagnosis of cancer can be waived for most medical co-payments by the NHI. This would reduce the detection bias related to social classes. Third, the potential bias related to self-reporting could be minimized by the use of medical records.

The study limitations included a lack of actual measurement data for potential confounders such as ionizing radiation, anthropometric factors, smoking, alcohol drinking, family history, lifestyle, diet, and genetic parameters. In addition, because of lack of information, it was not possible to evaluate the impacts of some biochemical and hormonal data such as glucose, insulin, C-peptide, lipid profile and thyroid-related hormones. Another limitation is the lack of information on the pathology, grading and staging of thyroid cancer. This would lead to a difficulty in analyzing the mechanism. Because papillary thyroid cancer represents 78.1% and 86.0%, in men and women, respectively, in the Taiwanese population [16], whether the findings of the present study could be related to this type of thyroid cancer and not limited to medullary thyroid cancer as previously suspected [1] awaits further confirmation. Finally, this study did not evaluate the effects of other DPP-4 inhibitors and GLP-1R agonists. Future studies are required to explore whether the findings can be extrapolated to other incretin-based therapies.

In summary, this study suggests that sitagliptin use among Taiwanese patients with type 2 diabetes mellitus may be associated with an increased risk of thyroid cancer, especially within the first year of its use. Therefore, new users of sitagliptin should be closely monitored for thyroid cancer development within the first year of its use. Future studies are required to confirm the findings.

## MATERIALS AND METHODS

Since March 1995 a unique, compulsory and universal health care system covering 99% of the residents, the so called NHI, has been implemented in Taiwan. Detailed description of the NHI databases can be seen elsewhere [17-21]. Diabetes was coded 250.XX and thyroid cancer 193, based on the *International Classification of Diseases, Ninth Revision, Clinical Modification* (ICD-9-CM).

Figure 1 shows the procedures in recruiting a cohort of patients with newly diagnosed type 2 diabetes mellitus during the period from 1999 to 2008 (original sample). Patients should have been followed after March 1, 2009 (the date of the approval for the reimbursement of sitagliptin by the Bureau of NHI) with 2 or more prescriptions of antidiabetic drugs in the outpatient clinic thereafter (*n* = 552484). To assure that diabetes was first diagnosed after 1999, patients who had a diagnosis of diabetes mellitus during 1996-1998 were not included. To avoid the contamination of other incretin-based therapies, users of saxagliptin (*n* = 3885), vildagliptin (*n* = 2502), and exenatide (*n* = 154) were excluded. Because incretin-based therapies were not approved for the treatment of type 1 diabetes mellitus, patients who held a Severe Morbidity Card certifying a diagnosis of type 1 diabetes were also excluded (*n* = 3243). A total of 511 patients were excluded because of missing data. Because incretin-based therapies might confer a potential risk of acute pancreatitis and some cancers like pancreatic cancer and thyroid cancer [2], patients who had been diagnosed at outpatient clinics or during hospitalization as having any cancer (*n* = 66841), or acute pancreatitis (*n* = 17792) before entry were excluded. Patients aged < 25 (*n* = 1687) or > 75 (*n* = 61604), and those who had been followed up for < 180 days (*n* = 26401) were also excluded.

Cumulative duration (months) of sitagliptin use was calculated from the reimbursement databases and tertiles of cumulative duration were used for analyses. A number of comorbidities and covariates were included as described in detail previously [17-21]: age, sex, diabetes duration, hypertension (ICD-9-CM code: 401-405), chronic obstructive pulmonary disease (a surrogate for smoking; 490-496), heart failure (398.91, 402.11, 402.91, 404.11, 404.13, 404.91, 404.93 and 428), nephropathy (580-589), eye disease (250.5, 362.0, 369, 366.41 and 365.44), dyslipidemia (272.0-272.4), stroke (430-438), ischemic heart disease (410-414), peripheral arterial disease (250.7, 785.4, 443.81 and 440-448), obesity (278) and benign thyroid disease (240-246). Other antidiabetic medications included sulfonylurea, metformin, meglitinide, acarbose, insulin, pioglitazone and rosiglitazone. Baseline characteristics between never users and ever users were compared by Student's t test for age and diabetes duration and by Chi-square test for other variables. The accuracy of disease diagnoses in the NHI database has been studied previously. Agreements between claim data and medical records are moderate to substantial, with Kappa values ranged from 0.55 to 0.86 [22].

The incidence density of thyroid cancer was calculated for never users and ever users and for different subgroups of exposure to sitagliptin. The numerator for the incidence was the number of patients with incident thyroid cancer during follow-up, and the denominator was the person-years of follow-up. Follow-up started on the first prescription of sitagliptin or comparators on or after March 1, 2009, and ended on December 31, 2011, at the time of a new diagnosis of thyroid cancer, or on the date of the last reimbursement record.

Logistic regression was used to create propensity score (PS) from the baseline characteristics. The treatment effect was estimated by using PS-weighting with the inverse probability of treatment weighting (IPTW) approach incorporated into a Cox regression [14]. Hazard ratios were estimated for ever users *versus* never users, and for each tertile of cumulative duration of sitagliptin therapy compared to never users as referent. Because metformin might potentially affect the risk of cancer, hazard ratios for ever *versus* never users of sitagliptin were also estimated for subgroups of metformin users and non-users, respectively. Because benign thyroid disease is a major risk factor for thyroid cancer, the above models were also created after excluding patients who had a diagnosis of benign thyroid disease at baseline.

To evaluate whether sitagliptin ever users and never users might have different screening rates of thyroid cancer, the prevalence of receiving thyroid sonography/aspiration examinations was compared between the two groups by Chi square test.

Imbalance in baseline characteristics between groups may lead to selection bias or residual confounding. To address these potential problems, additional analyses were conducted by using a 1:1 matched-pair sample based on 8 digits of PS according to the methods described by Parsons (matched sample) [23]. Austin and Stuart proposed a quantitative method as a formal test for balance diagnostics based on the calculation of standardized difference and recommended a value of > 10% as an indication of meaningful imbalance with potential confounding [24]. The standardized differences for all covariates were calculated as described by Austin and Stuart [24].

Analyses were conducted using SAS statistical software, version 9.3 (SAS Institute, Cary, NC). *P* < 0.05 was considered statistically significant.
